# Genetic subgroup of small ruminant lentiviruses that infects sheep homozygous for *TMEM154* frameshift deletion mutation A4^Δ^53

**DOI:** 10.1186/s13567-015-0162-7

**Published:** 2015-03-05

**Authors:** Michael L Clawson, Reid Redden, Gennie Schuller, Michael P Heaton, Aspen Workman, Carol G Chitko-McKown, Timothy PL Smith, Kreg A Leymaster

**Affiliations:** United States Department of Agriculture (USDA) Agricultural Research Service (ARS), U.S. Meat Animal Research Center (USMARC), State Spur 18D, Clay Center, NE, 68933 USA; Department of Animal Sciences, North Dakota State University, Fargo, ND 58108-6050 USA

## Abstract

Small ruminant lentivirus (SRLV) infections of sheep are influenced by genetics on both the host and pathogen sides. Genetic variation in the ovine transmembrane 154 (*TMEM154*) gene associates with infection susceptibility, and distinct SRLV genetic subgroups infect sheep in association with their *TMEM154* diplotypes. In this study, a novel SRLV subgroup was identified that naturally infected sheep with various *TMEM154* diplotypes, including those homozygous for a rare frameshift mutation (A4 delta53), which is predicted to abolish TMEM154 protein function. Thus, these SRLVs may infect sheep that lack functional TMEM154, and may not be restricted by *TMEM154* diplotypes in establishing infections.

**Electronic supplementary material:**

The online version of this article (doi:10.1186/s13567-015-0162-7) contains supplementary material, which is available to authorized users.

## Introduction, methods and results

Small ruminant lentiviruses (SRLVs) are genetically diverse retroviruses that infect monocytes, macrophages, dendritic cells, and microglial cells of domestic sheep, goats, and wild ruminants [1-4]. Infections persist for the lifetime of the host, causing chronic inflammation and a slow progression to disease [5,6]. Common symptoms of SRLV infection in sheep include interstitial pneumonia with dyspnea, indurative mastitis, and cachexia [7]. There are no available preventative vaccines or cures for SRLVs, and the disease affects sheep and goats in the U.S. and around much of the world [8-11].

In sheep, there is a strong genetic component to the relative risk of SRLV infection on both the host and pathogen sides [12,13]. On the host side, genetic variation in the ovine transmembrane 154 (*TMEM154*) gene associates with SRLV infection susceptibility [12,14]. The biological function of TMEM154 is unknown; however, it is predicted to be a type I membrane protein based on its amino acid sequence. Twelve non-synonymous SNPs have been detected in regions of *TMEM154* that encode predicted leader or extracellular domains of the protein, giving rise to 12 haplotypes that each encode different isoforms (Table 1) [12]. Haplotypes 1, 2, and 3 are the most common haplotypes found in sheep, and all three have an effect on SRLV susceptibility. Sheep with a copy of either haplotype 2 or 3, both of which encode a glutamate amino acid residue at position 35 (E35) of the extracellular portion of TMEM154, have an increased risk of SRLV infection. Conversely, sheep homozygous for haplotype 1, which encodes a lysine residue at position 35 (K35), have a decreased risk of infection (Table 1) [12,15].

**Table 1:** **Ovine** ***TMEM154*** **haplotypes and their associations with infection susceptibility and SRLV subgroups**

**Haplotype**	**Amino acid at position indicated**												**Allelic effect** ^**b**^	**SRLV association**
	**4** ^**a**^	**13**	**14**	**25**	**31**	**33**	**35**	**44**	**70**	**74**	**82**	**102**		
**1**	R	A	L	T	E	D	K	T	N	I	E	I	Less-susceptible	Subgroup 1
**2**	R	A	L	T	E	D	E	T	I	I	E	I	Highly-susceptible	Subgroup 2
**3**	R	A	L	T	E	D	E	T	N	I	E	I	Highly-susceptible	Subgroup 2
**4**	A	P^c^	F^c^	P^c^	R^c^	T^c^	N^c^	W^c^	NA^d^	NA	NA	NA	Unknown	Unknown, can be infected by Subgroup 4 (this study)
**6**	R	A	L	T	E	D	E	T	N	I	Y	NA	Unknown	Unknown
**9**	R	A	L	T	E	N	E	T	N	I	E	I	Unknown	Unknown
**10**	R	A	H	T	E	D	K	T	N	I	E	I	Unknown	Unknown
**11**	R	A	L	I	E	D	E	T	N	I	E	I	Unknown	Unknown
**12** ^**e**^	R	A	L	T	E	D	E	T	N	F	E	I	Unknown	Unknown
**13**	R	V	L	T	E	N	E	T	N	I	E	I	Unknown	Unknown
**14**	R	A	L	T	E	D	E	T	N	I	E	T	Unknown	Unknown
**15** ^**e**^	R	A	L	T	Q	D	E	T	N	F	E	I	Unknown	Unknown

^a^Numbers refer to amino acid positions in [GenBank:HM355886].

^b^Does not account for SRLV subgroups.

^c^Result of frameshift mutation at amino acid position 4.

^d^Not applicable due to preceding premature stop codon.

^e^Haplotype observed in wild sheep.

On the pathogen side, two SRLV subgroups defined by genetic variation within segments of proviral *gag* and *env* genes, associate with alleles of the ovine *TMEM154* E35K polymorphism [13]. SRLV subgroup 1 associates with homozygous and hemizygous *TMEM154* K35 genotypes with the K allele encoded by haplotype 1, and subgroup 2 associates with hemi-and homozygous *TMEM154* E35 genotypes with the E allele encoded by haplotypes 2 or 3 (Table 1) [13]. Thus, some SRLVs have adapted to infect sheep with distinct *TMEM154* E35K genotypes and can influence *TMEM154* E35K susceptibility to infection.

The biology responsible for *TMEM154* E35K associations with SRLV infection susceptibility, and SRLV subgroup associations with *TMEM154* E35K genotypes is unknown. While a portion of TMEM154 is predicted to extend from the host cell into the external milieu, it is not known if TMEM154 serves as a receptor, or co-receptor for SRLV attachment to the host cell, affects viral processing within the cell, or exit from it, or has a different biological function regarding SRLV infection. It is also not known if genetic variation in proviral *gag* and/or *env* directly causes SRLV subgroups to associate with *TMEM154* E35K genotypes, or is in linkage disequilibrium with causal alleles elsewhere in the SRLV genome. However, given that SRLV subgroups and *TMEM154* E35K genotypes associate with each other through apparent coevolution, TMEM154 is implicated as having a critical role in SRLV infections, leading us to hypothesize that sheep lacking functional TMEM154 may be completely resistant to SRLV infection.

*TMEM154 A4Δ53* is a frameshift mutation that is predicted to abolish protein function [12]. With leader peptide, the ovine TMEM154 precursor protein encoded by haplotypes 1, 2, and 3 is 191 amino acids, and yields a mature protein of 161 amino acids once the predicted leader peptide has been removed [12]. *TMEM154 A4Δ53* is part of haplotype 4, and encodes a single nucleotide deletion at amino acid position four of the precursor protein which results in an extensive change of amino acid coding at and downstream of amino acid four, and the first of multiple premature stop codons starting at amino acid position 54 (Table 1). Consequently, a truncated 53 amino acid protein with little homology to other TMEM154 isoforms results from the mutation, and sheep that are homozygous for *TMEM154 A4Δ53* constitute natural TMEM154 “knockouts”. Although generally rare, the *TMEM154 A4Δ53* allele has been found in multiple sheep breeds in the U.S., and in sheep from Turkey, Iran, Spain, France, and India [12,16]. Thus, sheep homozygous for *TMEM154 A4Δ53* are distributed throughout multiple geographical regions that may have their own distinct SRLV populations.

As part of a research surveillance program, a flock of approximately 250 sheep at North Dakota State University was sampled for SRLV infection and *TMEM154* diplotypes. The flock was comprised of multiple breeds including Columbia, Dorset, Hampshire, and Katahdin. Members of the flock were approved for research use by the animal care and use committee of the North Central Region-Sustainable Agriculture Research and Education program (Project Number FNC13-929). *TMEM154* diplotypes were scored using a commercially run matrix-assisted laser desorption/ionization-time-of-flight mass spectrometry (MALDI-TOF MS) assay (GeneSeek, Lincoln, NE, USA) [16], and SRLV infection status was determined serologically using a commercially run competitive enzyme linked immunosorbent assay (cELISA), (GeneSeek [17]). Over 50% of the flock was infected, including a Hampshire ewe and a Katahdin ewe that were both *TMEM154* “4,4” diplotypes, and thus homozygous for *TMEM154 A4Δ53* (Table 2).

**Table 2:** **SRLV-infected sheep sampled for proviral** ***gag*** **sequence**

**Animal ID**	**Breed**	**Age**	***TMEM154*** **diplotype**	**SRLV** ***gag*** **GenBank #**
6004	Dorset	7	1,1	KP120553
7006	Dorset	6	1,1	KP120547
7056	Columbia	6	1,1	KP120545
8344	Columbia	5	1,1	KP120540
9032	Columbia	4	1,1	KP120543
261	Dorset	3	1,2	KP120554
6012	Dorset	7	1,2	KP120549
7028	Dorset	6	1,2	KP120539
9451	Dorset	4	1,2	KP120541
256	Hampshire	3	1,4	KP120542
6542	Hampshire	7	1,4	KP120544
7434	Hampshire	6	1,4	KP120546
8539	Katahdin	5	1,4	KP120555
9322	Hampshire	4	1,4	KP120548
15	Hampshire	3	2,4	KP120552
1150	Hampshire	2	4,4	KP120550
8540	Katahdin	5	4,4	KP120551

The MALDI-TOF MS genotyping results for both of the *TMEM154* “4,4” ewes, were confirmed with Sanger sequencing of *TMEM154* DNA and cDNA amplified from their blood. The sequencing was conducted on PCR-generated amplicons of *TMEM154* exons 1 and 2, and *TMEM154* cDNA that was RT-PCR amplified with previously described reagents and conditions [12,16]. The sequences were produced with an ABI 3730 capillary sequencer (PE Applied Biosystems, Foster City, CA, USA) and assembled into complete contigs with Phred and Phrap [18,19], Polyphred (version 6.10), and Consed software [20], and visually genotyped. By DNA and cDNA sequence, both ewes were homozygous for *TMEM154 A4Δ53* and had *TMEM154* “4,4” diplotypes [GenBank: KP142216 - KP142219]*.* Thus, the *TMEM154* DNA and corresponding mRNA sequences were identical, with no evidence of alternate splicing of *TMEM154* at the transcript sequence level.

To verify the infection status of the two seropositive ewes with *TMEM154* “4,4” diplotypes, and to characterize the SRLVs they were infected by, nested PCR for SRLV proviral *gag* was conducted on blood DNAs isolated from the two animals (Table 2). Additionally, the PCR was conducted on blood DNAs from 15 seropositive ewes of the same flock with other *TMEM154* diplotypes, to determine if similar SRLVs had infected sheep with or without *TMEM154* “4,4” diplotypes (Table 2). Previously described reagents, conditions, and methods were used for the PCR [13]. The amplicons were Sanger sequenced and assembled with Phred, Phrap, Polyphred (version 6.10), and Consed software [18-20]. All 17 seropositive ewes were confirmed positive for SRLV infection by proviral *gag* amplification and sequence.

**Figure 1:**
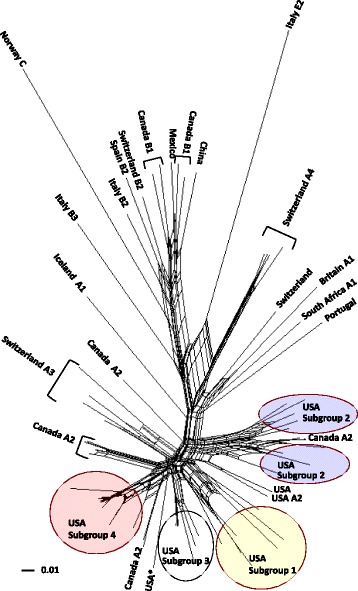
**Neighbor-Joining network of SRLV partial** ***gag*** **sequences.** The sequences were either produced in this study or were available from GenBank, and with amplification primer sites excluded correspond to nucleotide positions 1290–1771 of reference sequence [GenBank:NC_001452]. Capital letters and numbers following the country of origin represent genotypes and subtypes, respectively. The asterisk represents the one sequence [GenBank:KP120539] that originated from a ewe of the North Dakota State University flock that did not cluster within USA subgroup 4. The scale bar represents substitutions per site.

**Figure 2:**
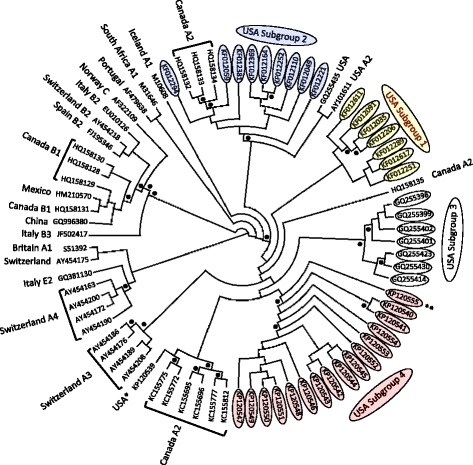
**Bootstrapped Neighbor-Joining tree of SRLV partial** ***gag*** **sequences.** The tree was produced from the same alignment used to produce the network in Figure 1. Like Figure 1, capital letters and numbers following the country of origin represent genotypes and subtypes, respectively. Outer taxonomic numbers represent GenBank accession numbers. Black dots represent bootstrap values greater than 80%. The single asterisk represents the one sequence that originated from an ewe of the North Dakota State University flock that did not cluster within USA subgroup 4. The double asterisk represents one sequence that clusters with subgroup 4 sequences but is not supported within the clade with a bootstrap equal to or greater than 80%. USA subgroups 1 and 2 both contain recombinant sequences that impact bootstrap support [13]. The tree is drawn as a circular cladogram and does not have a scale bar.

In addition to the recent classification of SRLV subgroups 1 and 2 that associate with sheep *TMEM154* E35K genotypes, SRLVs from around the world have been typed into major genotype groups A-E, and into subtypes within the genotype groups based on *gag* and/or *pol* variation [13,21-23]. To characterize the SRLVs infecting the North Dakota sheep, their *gag* sequences were compared to 1) those of SRLVs from North America and elsewhere in the world, and 2) the top ten closest blast matches to the *gag* sequence from one of the infected *TMEM154* “4,4” ewes in a Neighbor-Net phylogenetic network (Figure 1) and a Neighbor-Joining tree (Figure 2), (see Additional file 1 for sequence information). The network was constructed as SRLVs can have recombinant genomes [24,25], including those that comprise subgroups 1 and 2 [13], and the network accounted for recombinant sequences. The Neighbor-Joining tree was constructed to evaluate clade support with bootstraps, although Neighbor-Joining trees do not account for recombinant sequences. The network was constructed in SplitsTree (version 4.12.3) [26]. The tree was constructed in PHYLIP [27] using the programs Seqboot, Dnadist, Neighbor, and Consense, and viewed in Dendroscope [28]. Both the network and the tree were produced from a *gag* alignment generated with ClustalW in MacVector (version 12.0.6), with an F84 model of substitution and a transition/transversion ratio of two. The tree was bootstrapped with 1000 pseudoalignments. Sequence representatives of SRLV subgroups 1 and 2 were included in the analyses, as were those of what we describe as subgroup 3, which infected a flock of sheep in the Western United States [29], and has not been tested for an association with *TMEM154* genotypes.

Of the North Dakota proviral *gag* sequences, 16 of 17 sequences clustered together in both the Neighbor-Net phylogenetic network and the Neighbor-Joining tree, indicating that a distinct SRLV subgroup infected members of the flock (USA Subgroup 4, Figures 1 and 2). Additionally, within the Neighbor-Joining tree, 15 of 16 sequences within the clade were supported by a bootstrap value greater than 80%, delineating a clear genetic distinction of these strains from all others in the tree as a subgroup (USA Subgroup 4, Figure 2). The *gag* sequences from both of the infected *TMEM154* “4,4” diplotype ewes placed in this subgroup, as did the sequences from ewes with *TMEM154* “1,1”, “1,2”, “1,4”, and “2,4” diplotypes (Figure 2, Table 2). This demonstrates that SRLVs of subgroup 4 are not restricted to infecting only sheep with *TMEM154* “4,4” diplotypes. Both the network and the tree show that USA subgroups 1, 2, 3, and 4 either contain or are flanked by genotype A, subtype A2 sequences. Additionally, the Neighbor-Joining tree has two bootstrap values greater than 80% that collectively support the clustering of all four USA subgroups with A2 sequences, with subtype A3 sequences from Switzerland comprising a related but separate clade (Figure 2). This indicates that all four USA subgroups are members of the A2 subtype.

The one SRLV *gag* sequence from a member of the North Dakota flock that was distinct from subgroup 4 originated from an ewe with a *TMEM154* 1,2 diplotype that was born in 2007, most likely in South Dakota. This ewe was introduced into the North Dakota flock in 2009. It is possible that she was infected with an SRLV prior to her introduction to the North Dakota flock.

## Discussion

Subgroup 4 SRLVs can infect sheep with *TMEM154* “4,4” diplotypes that are homozygous for the *TMEM154 A4Δ53* mutation, as well as sheep that do not have haplotype 4. This indicates that subgroup 4 SRLVs do not require functional TMEM154 to infect sheep, and may be able to infect sheep with any *TMEM154* diplotype. The mechanism(s) or host receptors that subgroup 4 SRLVs use to infect sheep in the absence of functional TMEM154 are unknown. Additionally, whether or not other SRLV subgroups infect cells in the same manner as subgroup 4 members at low frequency is also unknown. Additional barriers to infection that are independent of the *TMEM154* locus may be required to prevent infection of sheep by subgroup 4 SRLVs.

All four SRLV subgroups that have been identified in the U.S. place within the A2 subtype. SRLV genotype A is highly diverse with 15 subtypes currently known [23]. To better understand the context of ovine *TMEM154* allele associations with SRLV infection susceptibility, it would be interesting to know if SRLV members of other A subtypes, genotypes B-E, and any circulating recombinant forms vary by an association with *TMEM154* alleles, including an ability to infect sheep with TMEM154 “4,4” diplotypes. Additionally, it would be beneficial to obtain full-length genomic sequences from members of the SRLV subgroups to identify viral alleles that are biologically responsible for *TMEM154* associations, potential interactions, or lack thereof. Ultimately, knowledge at this level could lead to precision control of ovine SRLV infection through genetic management of both the host and the pathogen.

## Additional file

Additional file 1:
***gag*** **sequences used for phylogenetic trees of Figures** 1 **and** 2 **.** Table containing the following information for the *gag* sequences used to construct the phylogenetic trees of Figures 1 and 2; GenBank accession number, genetic subgroup affiliation, phylotype, production in this study, country of origin.
